# Metabolism and Vascular Retinopathies: Current Perspectives and Future Directions

**DOI:** 10.3390/diagnostics12040903

**Published:** 2022-04-05

**Authors:** Charandeep Singh

**Affiliations:** Liver Center, Division of Gastroenterology, Mass General Hospital, Boston, MA 02114, USA; csingh3@mgh.harvard.edu

**Keywords:** metabolism, ROP, AMD, DR, HIF, mitochondria, retinal metabolism

## Abstract

The retina is one of the most metabolically active organs in the body. Although it is an extension of the brain, the metabolic needs of the retina and metabolic exchanges between the different cell types in the retina are not the same as that of the brain. Retinal photoreceptors convert most of the glucose into lactate via aerobic glycolysis which takes place in their cytosol, yet there are immense numbers of mitochondria in photoreceptors. The present article is a focused review of the metabolic dysregulation seen in retinopathies with underlying vascular abnormalities with aberrant mitochondrial metabolism and Hypoxia-inducible factor (HIF) dependent pathogenesis. Special emphasis has been paid to metabolic exchanges between different cell types in retinopathy of prematurity (ROP), age-related macular degeneration (AMD), and diabetic retinopathy (DR). Metabolic similarities between these proliferative retinopathies have been discussed.

## 1. Introduction

**Figure 1:**
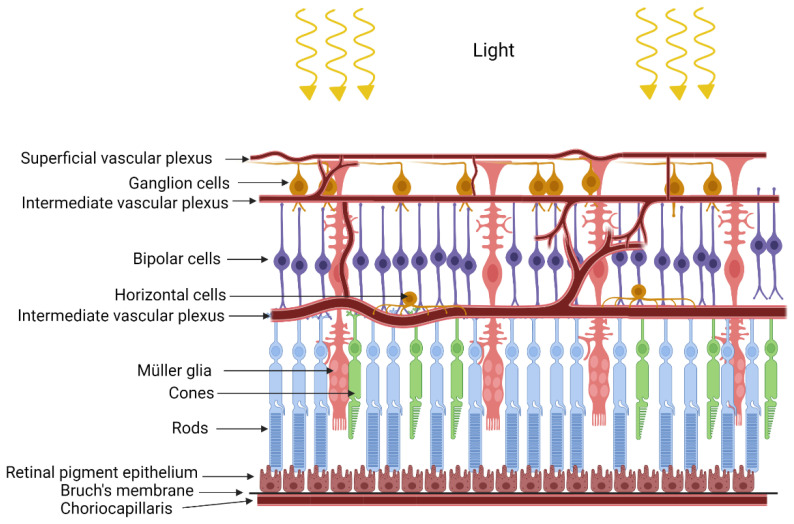
**Structure of the retina**. Depicted is the structure of the retina displaying layered structure of different cell types. The RPE separates choroidal blood supply from the retina. Müller cells span the whole length of the retina maintaining close metabolic connections between the inner retinal blood vessels and the photoreceptors (rods and cones).

The retina is formed by very well-organized layers of different types of cells with close metabolic symbiotic exchanges between these cell types (Figure 1). Retinal pigmented epithelium (RPE) serves as a barrier between the retina and choriocapillaris. Based on the blood supply distribution to the retina, retinas can be classified as vascular or avascular. The vascular retina is further subdivided in euangiotic or holangiotic pattern (humans, pigs, dogs), merangiotic (a smaller part of the retina like the rabbit), and paurangiotic (minute and restricted to the direct neighborhood of the optic disc, like the horse and the guinea pig). The avian retina is completely avascular (anangiotic pattern) [1]. Vascularized retinae have a dual blood supply mechanism, supplied with oxygen and nutrients through the retinal blood supply in addition to the choroidal blood supply. Blood vessels in the vascularized retina have tight junctions which serve as blood-retinal barrier to protect the retina from pathogens or toxins [2]. Unlike retinal vasculature, choroidal vasculature is fenestrated, but the retinal pigmented epithelium has tight junctions to form a blood-retinal barrier between the choroidal blood supply and the retina [3].

Retinal pigmented epithelium, retinal blood vessels, and neurons are metabolically coupled to the retinal macroglial Müller cells (Figure 1). Müller cells in the retina are specialized glial cells that span the whole length of the retina. Müller cells have multiple functions which include retinal repair, innate immune response, preventing retinal cell death by releasing neurotrophic factors, and releasing pro-inflammatory cytokines [4,5,6]. In terms of their metabolic role, Müller cells maintain retinal ion balance and pH. In addition, Müller cells are the sole producers of glutamine required for endothelial cell proliferation and to produce glutamate for neurotransmission in the retina. They also produce D-Serine for neurotransmission [6,7,8]. Mitochondrial localization in the Müller cells is different between vascular and avascular retinas [9]. Mitochondria are distributed along the full length of the Müller cells in vascularized retinas, and only present in close proximity to the choroid in the avascular retina, which implies oxygen-dependent distribution of the mitochondria in the Müller cell. In addition, mitochondria relocate and redistribute themselves in the isolated avascular retinal explants in culture where oxygen gradient is missing, implying oxygen dependent localization of Müller cell mitochondria [9].

The retina and RPE are mutually dependent on metabolic exchanges between them. RPE cell death leads to photoreceptor cell death in the retina, and vice versa [10]. RPE relies on proline from circulation for its metabolic needs and spares glucose for retinal use [11,12,13]. The retina is the only tissue with very high glycolytic demands. Unlike most of the other tissues, the purpose of the glycolytic pyruvate in the retina is to produce lactate, even in the presence of excessive oxygen [14,15,16,17]. This rapid conversion of glucose to lactate, also referred to as aerobic glycolysis, was first described by Otto Warburg [18]. Aerobic glycolysis primarily happens in the cytosol of the cells, and in the retina these enzymes localize primarily in photoreceptors, including all the enzymes responsible for converting glucose to pyruvate and the enzyme responsible for the final step of aerobic glycolysis—lactate dehydrogenase [19]. Contrary to what is expected from these findings, photoreceptors have high oxygen demand, and they have immense numbers of mitochondria [20]. Neither the oxygen nor the mitochondria are required for aerobic glycolysis, implying that the photoreceptors additionally rely on mitochondrial-dependent non-glycolytic metabolic pathways for their functioning. A substantial amount of energy in the retina is produced from non-glycolytic sources [17]. The tricarboxylic acid (TCA) cycle and oxidative phosphorylation (OXPHOS) are two mitochondrial interconnected pathways which actively produce energy in most of the non-cancerous cells. Fatty acid β-oxidation is one of the non-glycolytic sources of acetyl-CoA, precursor to mitochondrial TCA cycle and OXPHOS. Enzymes and receptors required for β-oxidation have been demonstrated to be present in the photoreceptor and Müller glia in the retina [21,22]. Mutations in fatty acid β-oxidation have been linked to retinopathies, which indicates that the mitochondrial metabolism is equally important as aerobic glycolysis for normal retinal function [21,23]. Given that mitochondria and mitochondrial metabolic pathways exist in the retina, it is imperative to look at these pathways in retinal diseases. At the transcriptional level, HIF1-α and HIF2-α regulate most metabolic processes which underlie angiogenesis [24,25,26]. The transcriptional response of HIF1-α and HIF2-α is reduced by HIF3-α [25,26,27]. Here we have reviewed a few recent publications where HIF-dependent mitochondrial metabolic pathways have been demonstrated to underlie the vasculopathies.

## 2. Retinopathy of Prematurity

Retinopathy of prematurity is a neurovascular retinal defect in prematurely born infants. Supplemental oxygen is needed to prevent mortality in the prematurely born infants with underdeveloped lungs [28]. On one hand, supplemental oxygen prevents motility, but on the other hand it suppresses development of the retinal blood vessel leading to hypovascularized retina in the phase I of ROP. The underdeveloped vasculature in phase I in the developing retina causes local hypoxia, due to limited supply of blood to the inner retina, leading to neovascularization in phase II of ROP [29,30,31,32]. There are several different animal models of oxygen-induced retinopathy (OIR) which can recapitulate neuro-vascular abnormalities seen in ROP [33]. Of all the models, mouse and rat models are used more often because they are economical, have large litter sizes, and are reproducible and robust models. The mouse OIR model has been used for a majority of the animal metabolic studies. All the animal models have the same underlying principle, i.e., animals are first subjected to a hyperoxic treatment which ceases blood vessel growth in the retina, followed by a return to normoxic room air causing local hypoxia in the retina which triggers retinal neovascularization [33,34]. Phase I and Phase II cause opposite effects on retinal vasculature development, and both these phases are expected to show complementary metabolic changes. Under normal development, new blood vessels are formed by proliferating and migrating endothelial cells. Proliferating endothelial cells have very high glutamine needs, and this need is fulfilled by the Müller cells [35,36]. Müller cells are the sole producers of retinal glutamine [37]. Baseline glutamine lyase (GLS) activity in the endothelial cells is higher than in any other cell type, implying that they are major consumers of glutamine. Although endothelial cells use glycolysis for their energy needs, they consume glutamine during development, which implies glycolysis in the endothelial cells is uncoupled from TCA.

In utero, blood vessels develop in a relatively hypoxic environment. Blood vessel development needs a hypoxic environment, where HIF-dependent pathways such as VEGF gradients, lactate gradients derived from the retinal neurons and Müller cells, play a central role in development [37,38]. All three, endothelial cells, neurons, and Müller cells, are dependent on each other for their metabolic needs during retinal vasculature development in the retina. In ROP and OIR models, hyperoxia degrades all isoforms of HIF-α by prolyl hydroxylase domain protein (PHD) dependent hydroxylation, a post-transitional modification, which requires molecular oxygen as one of the substrates [28,39]. Hydroxylation of HIF is followed by ubiquitination, which marks it for proteasomal degradation, whereas hypoxia stabilizes HIF and is beneficial for during the retina development [28]. Hypoxic preconditioning has been demonstrated to be beneficial in angiogenesis, to prevent neurons from ischemic injury, and to protect photoreceptors from light damage [40,41,42]. An alternative strategy to stabilize HIF, by PHD inhibition using pharmacological inhibitor dimethyloxallyl glycine (DMOG), has been applied in cardio-protection in ischemic injury models [43]. Hepatic HIF stabilization by PHD inhibition either by DMOG or FG-4592 has been demonstrated to be protective against phase I of OIR in rat and mouse models [44,45,46]. Hepatic HIF1 knockout (KO) mice are not protected against OIR when treated with DMOG, implying that DMOG acts via hepatic HIF dependent pathways [45].

To understand the metabolic basis of OIR and protection in HIF stabilized condition, we performed an untargeted metabolite profiling of polar metabolites extracted from the retina or the plasma of the P10 old noromoxic, hyperoxic, and hyperoxic mice treated with HIF stabilizer FG-4592 [28]. We found increased serine/one-carbon pathway flux in hyperoxic mice treated with FG-4592. Serine/one-carbon pathway provides biosynthetic precursors such as nucleic acids, NADPH for lipogenesis, and s-adenosylmethonine for methylation reactions to the developing cells in the retina. In addition, we also found systemic upregulation of proline and urea cycle metabolites in OIR mouse models treated with HIF stabilizer FG-4592. Moreover, using ^13^[C]_6_ glucose, we demonstrated that the retinal explant from P10 age mice were unable to produce their own serine or glycine, and retinal serine at this age is derived from circulation. Inhibition of serine/one-carbon increased avascular area in the retinas treated with FG-4592 as compared to controls [28]. Paris et al. analyzed metabolic difference in the ocular metabolome (whole eye) of the OIR model mice vs. nomoxic controls, in the phase II at P12, P14, and P17. Arginine and interconversion to proline, urea cycle, and β-oxidation were also demonstrated to be upregulated in the whole eye metabolome of the OIR phase II mouse model, whereas purine pathway metabolites were found to be downregulated [47]. These findings together reflect downregulation of biosynthetic metabolites necessary for normal growth of the retina in phase I and upregulation of these pathways in proliferative phase II of OIR. Tomito et al. analyzed metabolites in the vitreous humor from proliferative diabetic retinopathy patients and retinal metabolites from P17 old OIR model hypoxic phase II of the mouse model. They found decreased levels of creatine and increased levels of glycine, in both the Proliferative Diabetic Retinopathy (PDR) patient vitreous samples and hypoxic P17 mouse retina. Creatine supplementation reduced OIR-linked neovascularization (NV) in the mouse model via downregulation of Vegf-a and Pdgf-b [48]. The creatine production pathway involves production of guanidinoacetate from arginine and glycine. By the action of Guanidinoacetate N-methyltransferase (GAMT) enzyme, guanidinoacetate and s-adenosylmethioine (SAM) produce creatine. The SAM required for this step is produced through carbon donated from serine in the one-carbon pathway. In the absence of SAM or inhibition of GAMT, one would expect to see substrates upstream of the enzyme, such as guanidinoacetate, glycine, and arginine, to accumulate. Zhou et al. performed a time-series comparative untargeted metabolomics analysis on the retinas from OIR model mouse and room-air controls at P12 (postnatal day 12), P13, P17, and P42. Many amino acids were found to be perturbed, confirmed by a targeted amino acid measurement. They found downregulation of many amino acids in P12 OIR mouse retinas which were non-significant, and after removal of mice to room air from P13-P42, amino acids were statistically significantly upregulated [49]. In a separate study, Zhaou et al. also screened plasma metabolome of the treatment requiring ROP patients and aged-matched controls. They found significant differences in the “aminoacyl-tRNA biosynthesis” and “protein digestion and absorption”, and these pathways were enriched via KEGG analysis of the untargeted metabolomics data. Using variable importance in projection in multivariate statistical analysis, they found 29 and 23 metabolite pool size differences in positive and negative ionization modes, respectively. However, using univariate comparisons of the metabolomics data, they found only 11 metabolites in positive and 13 metabolites in negative ionization modes to be different with the univariate comparison [50].

All these recent studies indicate perturbation in amino acid metabolism, specifically serine/one-carbon metabolism, in arginine and proline, and nitrogen metabolism in the ROP or OIR. Of these metabolic pathways, arginine metabolism to ornithine by arginase has been studied earlier in a neurodegeneration context in retinal OIR models. Mitochondrial isoform of arginase, arginase 2, has been shown to be involved in apoptosis of neurons in the mouse OIR model [51]. Ornithine, produced by arginase reaction, is utilized to produce polyamines and can additionally be used for proline synthesis, both of which are important biomass precursors. The mechanism underlying arginase 2 deficiency being beneficial in OIR models has been shown to involve excessive production of polyamine and oxidation of polyamine products to their precursor generating toxic side products. Arginase 2 activity has been localized mainly to horizonal cells in the retina. Narayanan et al. demonstrated increased expression of polyamine oxidation enzyme spermine oxidase (SMO) in response to hyperoxia. Toxic side products from polyamine breakdown, such as acrolein, 3-acetaminopropanol, 3-aminopropanol, and H_2_O_2_, have been implied to underlie the neurotoxic apoptotic activity in the OIR retina [51].

We demonstrated that hyperoxia downregulates proliferation of human primary retinal endothelial cells in the culture. We have also seen upregulation of polyamine degradation enzymes SMOX and PAOX, and decreased levels of putrescine in hyperoxic treated primary endothelial cells. We demonstrated downregulation of Myc in response to hyperoxia [52]. Myc and HIF have very overlapping control over biosynthetic pathways. Ornithine decarboxylase is one of the Myc targets, and ODC has been demonstrated to peak at the G1-S and G2-M phase of the cell cycle [53]. This demonstrates that metabolic signaling by polyamine metabolites triggers cell cycle arrest in endothelial cells. In addition, we also demonstrated that hyperoxia alters glutamine metabolism in retinal Müller cells. Hyperoxia blocks entry of glycolytic flux into TCA and in response to which Müller cells start to use glutamine for their anaplerotic needs [54]. Cultured primary and immortalized Müller cells exposed to hyperoxic conditions lose this property and can no longer assimilate ammonium into glutamine [54]. Müller cells are the sole producers of glutamine, and in hyperoxic conditions we saw a reversal of this function to glutamine utilization rather the production. These alterations probably explain why, in many studies, urea cycle metabolites such as arginine, ornithine, and proline were found to be altered.

Other than the central carbon metabolism discussed above, polyunsaturated fatty acids, arachidonic acid, and Docosahexaenoic Acid (DHA) have been shown to be low in the blood of preterm infants who develop ROP [55,56,57,58]. The mouse model of OIR treated with DHA improves, and Arachidonic acid (AA) exacerbates, neovascularization [59,60].

**Figure 2:**
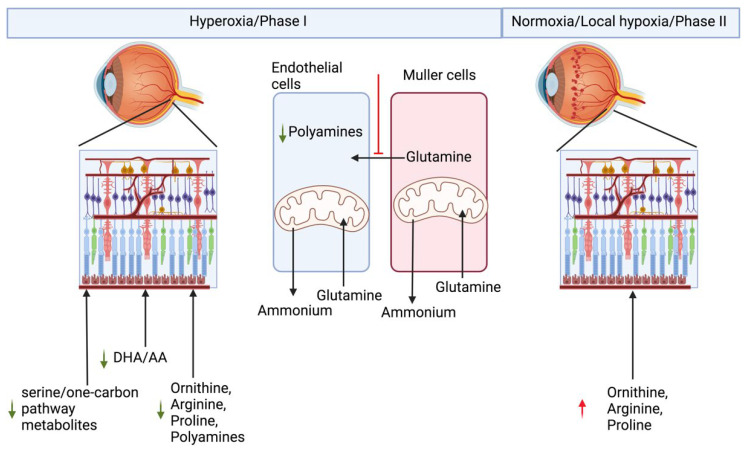
**Metabolic basis of retinopathy of prematurity**. Hyperoxia downregulates biosynthetic pathways leading to hypovascularized retina in phase 1 of ROP/OIR. Hypovascularized retinas, when exposed to room air in the phase 2 of ROP/OIR, lead to aggressive upregulation of neovascularization in the retina, leading to blindness.

Altogether, all the studies point toward a systemic metabolic imbalance underlying ROP development, and a systemic biochemical multi-organ metabolic model is much needed to understand and find treatment for ROP. Biochemical alterations seen in OIR/ROP have been summarized in the Figure 2.

## 3. Age-Related Macular Degeneration (AMD)

**Figure 3:**
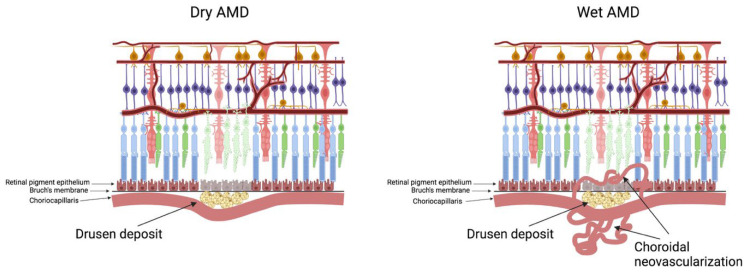
**Age-related macular degeneration.** There are two types of AMD, dry and wet. In dry-AMD, drusen deposits in the between choriocapllaris and RPE, blocking flow of metabolites and oxygen. In the later stage wet-AMD, hypoxia due to oxygen gradient and HIF stabilization leads to neovascularization and loss of central vision.

Retinal pigmented epithelium (RPE) degeneration leads to photoreceptor death in the AMD, causing vision loss in the macular region of the retina. There are two different types of AMD: wet-AMD and dry-AMD (Figure 3). In the dry-AMD, drusen deposits between the retina and the choroid, leading to blockage in the metabolic transport to the retina, whereas the more aggressive later stage, the wet-AMD, is characterized by choroidal neo-vascularization, which affects about 10–15% of the total cases of AMD yet accounts for the major cause of vision loss [61]. Drusen proteome and metabolome have been studied by many labs. Craab et al. (2002) studied proteome of drusen in AMD patients and identified TIMP3, serum albumin, vitronectin, and clusterin as the most frequently found proteins in drusen [62]. Interestingly, mutation in the TIMP3 gene has been linked to the AMD-like disease Sorsby fundus dystrophy (SFD) [63]. TIMP3 has been demonstrated to inhibit the VEGF-dependent angiogenic pathway and prevent neovascularization seen in SFD. Like AMD, drusen-like deposits are seen in SFD. While drusen is an extracellular deposit seen in AMD patients, lipofusin deposit is seen intracellularly in RPE cells. Lipofuscin is not seen in age-related diseases exclusively. Batten disease (neuronal ceroid lipofuscinoses) is a rare genetic disease which mostly affects children with mutations in their lysosomal enzymes where ceroid lipofuscin deposits are seen, similar to that seen in the AMD [64]. The ceroid lipofuscin deposits seen in Batten disease contain very high amounts of mitochondrial ATPase subunit c along with fatty acids [65]. Several units of ATPase have been seen in the human ocular ceroid-lipofuscin, indicating a mechanistic failure of mitochondria in these pathologies [65].

RPE acquires its nutrient supply from the blood supplied by choriocapillaris, and tight junctions between RPE cells serve as a barrier between blood supply and the retina [10]. RPE highly depends on proline for its own metabolic needs and spares other nutrients such as glucose, serine, and glutamine for retinal use [11,66]. Proline is either acquired from blood or is produced from the collagen broken down locally in the RPE. Proline can serve as anaplerotic substrate to produce energy via OXPHOS, where the first step is catalyzed by proline dehydrogenase (PRODH). This process produces glutamate, which enters at α-ketoglutarate into TCA cycle. Interestingly, unlike most of the mitochondrial rich normal cells in the body, RPE utilizes reductive carboxylation instead of oxidative decarboxylation [67]. Photoreceptor health depends highly on RPE metabolism and transport of nutrients from the RPE to the retina. It has been shown in multiple experiments that any changes in RPE health affect retinal health in long run [10]. Aberrant metabolism of both the RPE and retina contribute to AMD development and progression. Recent studies point towards mitochondrial metabolic abnormalities underlying the development and progression of the disease. Lain et al. performed pathway enrichment analysis of plasma metabolome of AMD patients, and they found aberrant “taurine and hypotaurine metabolites”, “sphingolipids metabolites”, “purine metabolites”, “glycerophospholipids”, and “nitrogen metabolites”. They also compared metabolome with disease progression and found additional pathways such as “citrate TCA metabolites”, “serine/one-carbon”, “arginine and proline metabolites”, “beta-alanine metabolites”, “panthothenate and CoA metabolites”, and “pyrimidine metabolites” [68]. Most of these anabolic pathways are assembled in mitochondria. These data reflect the idea that systemic mitochondrial abnormalities may underlie progression of AMD. Michael et al. also compared the plasma metabolome of patients with different degrees of AMD to that of controls. They saw higher amounts of acylcarnitines in neovascular AMD patient group as compared to the dry-AMD patient group. In addition, they also saw few phospholipids, ethanolamine derivatives of lipids, steroids, and pyroglutamate in AMD patients compared to controls [69]. The EYE-RISK Consortium conducted the largest ever metabolomics study where they performed comparative plasma metabolomics of 2307 patients and 4294 controls. Excluding known biomarkers, 60 metabolites were found to be linked to AMD. They found VLDL, HDL lipoproteins, apolipoproteins, amino acids, citrate, and fatty acids. Overall, HDL levels were higher and VLDL levels were lower in AMD patients. Associations were found between lipid metabolism genes APOE, LIPC, CETP, and ABCA1 with metabolomics data [70]. Apolipoproteins have also been implicated in AMD. Consensus from the published data demonstrates APOE2 predisposes carriers to AMD, whereas APOE4 is protective against AMD. However, both APOE2 and APOE4 carriers are predisposed to Alzheimer’s disease. APOE is an important component of VLDL and LDL secreted from the liver. LDL receptors on the cells sense APOE on LDL to utilize LDL components. APOE is produced by RPE and also produced by retinal Müller macroglial cells. Mouse models with mutations in LDL receptors show promise as models to study AMD in vivo [71]. Using this model may help better understand the factor underlying the development of AMD in humans.

## 4. Diabetic Retinopathy

Diabetic retinopathy is an example of systemic pathogenesis, where uncontrolled systemic hyperglycemia underlies the damage to the retinal blood vessels. Diabetic vascular retinopathy can be divided into two sub-types based on progress in the disease development. Early stage is referred to as non-proliferative DR, which is characterized by blood vessel damage and loss. Severe microvascular loss to the blood vessels supplying nutrients to the retina leads to neuronal damage and causes local hypoxia in the retina. This local hypoxia initiates the more aggressive stage of the DR, referred to as proliferative DR, in which neovascularization takes place. Sustained hyperglycemia leads to buildup of sorbitol by the action of aldose reductase [72]. Sorbitol, a sugar alcohol or polyol, causes osmotic stress in the retina, thereby further damaging blood vessels in the retina. Sustained systemic hyperglycemia also results in non-enzymatic spontaneous glycation of proteins which are referred to as advanced glycation end products (AGE). Diabetes leads to the breakdown of the blood-retinal barrier, leading to leaky blood vessels. Leaky vasculature allows accumulation of fluid in the retina and consequently leads to macular edema, causing vision loss in the macular region of the retina. Apart from the vascular component of the disease, other cells such as Müller cells contribute to the neurovascular component of the pathology. Diabetes induces GFAP expression in the Müller cells which leads to gliosis [73].

There are ample metabolic studies on DR patients published lately, and it is out of the scope of this manuscript to discuss them all in this review. Only a few of the latest research articles have been discussed here. Tomita et al. compared vitreous metabolome from PDR patients to non-diabetic controls. They found increased concentrations of lactate, pyruvate, allantoin, and proline in PDR patients compared to non-diabetic controls. Pathway enrichment analysis demonstrated changes in serine, glycine, proline, and arginine pathways. They also found increased concentrations of glycine and decreased concentration of downstream product creatine [48]. Yun et al., measured metabolic changes in the plasma of non-proliferative and proliferative DR and compared it the non-diabetic controls. They found 16 metabolites which separated DR patients from non-diabetic controls. Kynurenine, tryptophan, and dimethyl arginine were demonstrated to be markers differentiating DR progression in the patients [74]. Zhu et al. compared the plasma metabolome of PDR patients to that of the DR patients with ≥10 years of diabetes without DR retinopathy [75]. They found four reliable PDR plasma biomarkers: fumaric acid, acetic acid, cytidine, and uridine [75]. Another study by Sumarriva et al. compared plasma metabolome of Non-proliferative diabetic retinopathy (NPDR), PDR, and non-diabetic controls. They found alterations in “arginine and citrulline-related pathways” in DR compared to non-DR controls. Additionally, Carnitine was found to be different between NPDR and PDR groups, implying alterations in fatty acid metabolism with disease progression [76]. Peters et al. performed a targeted screening of arginine, asymmetric dimethylarginine, proline, ornithine, arginosuccinate, and citrulline, using isotope dilution mass-spectrometry in the plasma metabolome of diabetic controls, NPDR and PDR. All four metabolites were found to be increased in the citrulline, arginine, arginosuccinate, ADMA, and ornithine in DR vs. diabetic controls. However, after multiple testing and thorough statistical adjustments for false positives, only arginine and citrulline were found to be elevated in type 2 DR patients [77]. Sun et al. compared plasma metabolome of DR and PDR patients and found differences in four metabolites: N-acetyltryptophan, glutamate, pseudouridine, and leucylleucine in these patients [78].

There are many different animal models to study DR. It can be induced by complete or partial removal of the pancreas or damage caused by certain drugs targeting pancreases. DR can also be induced by a diet high in galactose or glucose, but these diet-induced models are used less frequently. Oxygen-induced retinopathy models are also used as a proxy of advanced PDR. There are also genetic models, like Akita (Insulin mutations leading to loss of beta cells and insulin production mimicking type I diabetes), db/db (Leptin Receptor mutation leading to obesity and diabetes), Kimba (trVEGF029) and Akimba (cross between Akita and Kimba). The most used DR models are streptozotocin-induced, db/db and Akita mice. Mora-Ortis et al. performed NMR-based metabolomics of db/db mouse model tissues, including of the eye. They found increased amounts of lipids and glucose in the eyes of db/db mice. Metabolites which were found to be decreased in the eyes of db/db mice were citrulline, alanine, glutamate, GABA, glutamine, hypoxanthine, isocitrate, myo-inositol, phenylalanine, O-phosphocholine, tyrosine, and inosine [79]. Patrick et al. used STZ-induced DB rat models and demonstrated changes in NADPH/NADP+, ATP/ADP, ADP/AMP, FADH2, tyrosine, citrulline, glutamine, methionine, leucine, aspartate, xanthine, pyridoxal phosphate, 4-aminobutanoate, riboflavin, 2-phosphoglycerate, PEP, 2-oxoglutarate, glycerone phosphate, sorbitol, acetyl CoA, glyoxylate, succinate, HCO_3_^−^, and CO_2_ [80].

## 5. Conclusions and Future Directions

Retinal vasculature is very sensitive to availability of nutrients, metabolic exchanges between cell types within and outside of retina, and oxygen availability. Hypoxia stimulates proliferation and migration of endothelial cells, which is seen in all the retinal vascular metabolic disorders discussed here. All three diseases discussed here in this review have common HIF-dependent pathogenesis, where hypoxia causes neovascularization in later stages. Overall, amino acid metabolism, serine/one-carbon, nitrogen metabolism, urea cycle, and fatty acid metabolism have been seen to underlie the development of these disorders. Recent findings demonstrate the systemic nature of these diseases and dependence on systemic metabolic changes.

Metabolomics in retinal research is still in a naïve stage. After the brain, the retina has the highest heterogeneity of cell types. The retina has six major classes of cells with a total of 58 cell types in the foveal region and 57 in the peripheral region [81]. Metabolic exchanges between the retinal cell types are not very well understood, as it is hard to differentiate metabolic signals from one cell from the other in the whole retinal metabolomics. The retina is one of the best organs to study vascular development in animal models because of the ease of visualizing retina from cornea. Metabolic exchanges between the cell types have been studied either in vivo or have been inferred from density of metabolic enzymes measured by immunohistochemical localization or immunocytochemical methods. However, it is very difficult to separate metabolic signals from one cell to another. At the cellular level, many enzyme and metabolic fluxes are controlled by posttranslational modification of enzymes. Although protein localization data is useful in finding out which pathways are abundant in one cell type versus another, it cannot inform us of active protein versus inactive protein in different cell types. The final layer of action in the cell is the time-sensitive metabolome. Even if there is an abundant amount of active enzyme, substrate to product conversion depends on substrate availability, and the amount of product should be lower than the concentration where it can inhibit the enzyme. More studies are needed at the cellular level to discern the metabolic contribution of each cell type in the retinal dysfunction. This involves developing better methods to culture different cells types in vitro. A more lucrative way to investigate these metabolic differences is to generate retinal organoids deficient in a certain cell type and study metabolic differences between a normal and mutated version. Such organoids have been used to study the function of retinal ganglion cells which cannot be cultured when isolated from the retina [82]. Kim et al. prepared retinal human organoids rich in cone cells to create an environment like human macula [83]. In the near future, organoid culture will offer options to study diseases which cannot be recapitulated in mouse models because of the absence of macula or difficulty in obtaining a specific gene knockout in retinal cell types of interest. It will also offer a better way to delineate metabolic signals specific to one or the other cell line. This system is ideal for studies where primary cells cannot be isolated at a scale good enough to study metabolic functions in vitro.

Many of these studies cited here relied on very limited metabolic pathways specifically because most of the labs measure polar metabolome, and lipidomic at the moment is not a routine method of choice in many labs. To have a better picture of the metabolic changes underlying these vascular retinopathies, one must study both the polar metabolome and the lipidome. Additionally, modalities of mass spectrometry like spatial metabolomics and imaging mass spectrometry may provide better insight into these metabolic retinopathies. Metabolome is a good starting point to find out aberrant pathways in these diseases; however, stable-isotope labeling of metabolites offers a better time-resolved metabolic change which cannot be obtained from single time-point metabolomics.

In summary, metabolomics is a strong tool to not only find biomarkers, but to also understand the metabolic basis of vascular retinopathies. It is encouraging to see similarities between metabolic findings between patient data sets and corresponding animal models in the publications cited in this review. Our current knowledge is limited to known biochemicals in metabolomic libraries or databases. However, the biggest problem with metabolomics is annotation of unknown compounds, and this limits the identification of novel metabolic markers or biochemicals. Combing untargeted metabolomics with stable isotope labeling of metabolites and machine learning may help find new biomarkers and metabolic causes to these vascular retinopathies.

## References

[1] De Schaepdrijver L., Simoens P., Lauwers H., De Geest J.P. (1989). Retinal vascular patterns in domestic animals. Res. Vet. Sci..

[2] Gardner W.T., Antonetti D.A., Barber A.J., LaNoue K.F., Levison S.W. (2002). Diabetic Retinopathy: More Than Meets the Eye. Surv. Ophthalmol..

[3] Erickson K.K., Sundstrom J.M., Antonetti D.A. (2007). Vascular permeability in ocular disease and the role of tight junctions. Angiogenesis.

[4] Goldman D. (2014). Müller glial cell reprogramming and retina regeneration. Nat. Rev. Neurosci..

[5] Kumar A., Pandey R.K., Miller L.J., Singh P.K., Kanwar M. (2013). Muller glia in retinal innate immunity: A perspective on their roles in endophthalmitis. Crit. Rev. Immunol..

[6] Bringmann A., Wiedemann P. (2012). Müller Glial Cells in Retinal Disease. Ophthalmologica.

[7] Bringmann A., Pannicke T., Grosche J., Francke M., Wiedemann P., Skatchkov S.N., Osborne N.N., Reichenbach A. (2006). Müller cells in the healthy and diseased retina. Prog. Retin. Eye Res..

[8] Bringmann A., Reichenbach A. (2001). Role of Muller cells in retinal degenerations. Front. Biosci. Landmark.

[9] Germer A., Biedermann B., Wolburg H., Schuck J., Grosche J., Kuhrt H., Reichelt W., Schousboe A., Paasche G., Mack A.F. (1998). Distribution of mitochondria within Müller cells—I. Correlation with retinal vascularization in different mammalian species. J. Neurocytol..

[10] Hurley J.B. (2021). Retina Metabolism and Metabolism in the Pigmented Epithelium: A Busy Intersection. Annu. Rev. Vis. Sci..

[11] Du J., Zhu S., Lim R.R., Chao J.R. (2021). Proline metabolism and transport in retinal health and disease. Amino Acids.

[12] Swarup A., Samuels I.S., Bell B.A., Han J.Y.S., Du J., Massenzio E., Abel E.D., Boesze-Battaglia K., Peachey N.S., Philp N.J. (2019). Modulating GLUT1 expression in retinal pigment epithelium decreases glucose levels in the retina: Impact on photoreceptors and Müller glial cells. Am. J. Physiol. Cell Physiol..

[13] Yam M., Engel A.L., Wang Y., Zhu S., Hauer A., Zhang R., Lohner D., Huang J., Dinterman M., Zhao C. (2019). Proline mediates metabolic communication between retinal pigment epithelial cells and the retina. J. Biol. Chem..

[14] Chinchore Y., Begaj T., Wu D., Drokhlyansky E., Cepko C.L. (2017). Glycolytic reliance promotes anabolism in photoreceptors. Elife.

[15] Winkler B.S. (1981). Glycolytic and oxidative metabolism in relation to retinal function. J. Gen. Physiol..

[16] Hurley J.B., Lindsay K.J., Du J. (2015). Glucose, lactate, and shuttling of metabolites in vertebrate retinas. J. Neurosci. Res..

[17] Cohen L.H., Noell W.K. (1960). Glucose catabolism of rabbit retina before and after development of visual function. J. Neurochem..

[18] Warburg O. (1956). On the Origin of Cancer Cells. Science.

[19] Lowry O.H., Roberts N.R., Schulz D.W., Clow J.E., Clark J.R. (1961). Quantitative Histochemistry of Retina: II. Enzymes of Glucose Metabolism. J. Biol. Chem..

[20] Lowry O.H., Roberts N.R., Lewis C. (1956). The quantitative histochemistry of the retina. J. Biol. Chem..

[21] Joyal J.S., Sun Y., Gantner M.L., Shao Z., Evans L.P., Saba N., Fredrick T., Burnim S., Kim J.S., Patel G. (2016). Retinal lipid and glucose metabolism dictates angiogenesis through the lipid sensor Ffar1. Nat. Med..

[22] Atsuzawa K., Nakazawa A., Mizutani K., Fukasawa M., Yamamoto N., Hashimoto T., Usuda N. (2010). Immunohistochemical localization of mitochondrial fatty acid β-oxidation enzymes in Müller cells of the retina. Histochem. Cell Biol..

[23] Heckel E., Cagnone G., Agnihotri T., Cakir B., Das A., Kim J.S., Kim N., Lavoie G., Situ A., Pundir S. (2022). Triglyceride-derived fatty acids reduce autophagy in a model of retinal angiomatous proliferation. JCI Insight.

[24] Hu C.-J., Wang L.-Y., Chodosh L.A., Keith B., Simon M.C. (2003). Differential roles of hypoxia-inducible factor 1alpha (HIF-1alpha) and HIF-2alpha in hypoxic gene regulation. Mol. Cell. Biol..

[25] Pugh C.W., Ratcliffe P.J. (2003). Regulation of angiogenesis by hypoxia: Role of the HIF system. Nat. Med..

[26] Wong B.W., Marsch E., Treps L., Baes M., Carmeliet P. (2017). Endothelial cell metabolism in health and disease: Impact of hypoxia. EMBO J..

[27] Carmeliet P. (2005). Angiogenesis in life, disease and medicine. Nature.

[28] Singh C., Hoppe G., Tran V., McCollum L., Bolok Y., Song W., Sharma A., Brunengraber H., Sears J.E. (2019). Serine and 1-carbon metabolism are required for HIF-mediated protection against retinopathy of prematurity. JCI Insight.

[29] Hartnett M.E., Lane R.H. (2013). Effects of oxygen on the development and severity of retinopathy of prematurity. J. Aapos.

[30] Smith L.E. (2003). Pathogenesis of retinopathy of prematurity. Semin. Neonatol..

[31] Smith L.E. (2008). Through the eyes of a child: Understanding retinopathy through ROP the Friedenwald lecture. Investig. Ophthalmol. Vis. Sci..

[32] Hartnett M.E., Penn J.S. (2012). Mechanisms and Management of Retinopathy of Prematurity. N. Engl. J. Med..

[33] Madan A., Penn J.S. (2003). Animal models of oxygen-induced retinopathy. Front. Biosci..

[34] Smith L.E., Wesolowski E., McLellan A., Kostyk S.K., D’Amato R., Sullivan R., D’Amore P.A. (1994). Oxygen-induced retinopathy in the mouse. Investig. Ophthalmol. Vis. Sci..

[35] Kim B., Li J., Jang C., Arany Z. (2017). Glutamine fuels proliferation but not migration of endothelial cells. EMBO J..

[36] De Bock K., Georgiadou M., Carmeliet P. (2013). Role of endothelial cell metabolism in vessel sprouting. Cell Metab..

[37] Newman E., Reichenbach A. (1996). The Müller cell: A functional element of the retina. Trends Neurosci..

[38] Joyal J.-S., Gantner M.L., Smith L.E.H. (2018). Retinal energy demands control vascular supply of the retina in development and disease: The role of neuronal lipid and glucose metabolism. Prog. Retin. Eye Res..

[39] Tojo Y., Sekine H., Hirano I., Pan X., Souma T., Tsujita T., Kawaguchi S.-I., Takeda N., Takeda K., Fong G.-H. (2015). Hypoxia Signaling Cascade for Erythropoietin Production in Hepatocytes. Mol. Cell. Biol..

[40] Akita T., Murohara T., Ikeda H., Sasaki K., Shimada T., Egami K., Imaizumi T. (2003). Hypoxic preconditioning augments efficacy of human endothelial progenitor cells for therapeutic neovascularization. Lab. Investig..

[41] Gidday J.M., Fitzgibbons J.C., Shah A.R., Park T.S. (1994). Neuroprotection from ischemic brain injury by hypoxic preconditioning in the neonatal rat. Neurosci. Lett..

[42] Grimm C., Wenzel A., Groszer M., Mayser H., Seeliger M., Samardzija M., Bauer C., Gassmann M., Remé C.E. (2002). HIF-1-induced erythropoietin in the hypoxic retina protects against light-induced retinal degeneration. Nat. Med..

[43] Eckle T., Köhler D., Lehmann R., Kasmi K.C.E., Eltzschig H.K. (2008). Hypoxia-Inducible Factor-1 Is Central to Cardioprotection. Circulation.

[44] Sears J.E., Hoppe G., Ebrahem Q., Anand-Apte B. (2008). Prolyl hydroxylase inhibition during hyperoxia prevents oxygen-induced retinopathy. Proc. Natl. Acad. Sci. USA.

[45] Hoppe G., Yoon S., Gopalan B., Savage A.R., Brown R., Case K., Vasanji A., Chan E.R., Silver R.B., Sears J.E. (2016). Comparative systems pharmacology of HIF stabilization in the prevention of retinopathy of prematurity. Proc. Natl. Acad. Sci. USA.

[46] Singh C., Sharma A., Hoppe G., Song W., Bolok Y., Sears J.E. (2018). 3-Hydroxypyruvate Destabilizes Hypoxia Inducible Factor and Induces Angiostasis. Investig. Ophthalmol. Vis. Sci..

[47] Paris L.P., Johnson C.H., Aguilar E., Usui Y., Cho K., Hoang L.T., Feitelberg D., Benton H.P., Westenskow P.D., Kurihara T. (2015). Global metabolomics reveals metabolic dysregulation in ischemic retinopathy. Metabolomics.

[48] Tomita Y., Cagnone G., Fu Z., Cakir B., Kotoda Y., Asakage M., Wakabayashi Y., Hellström A., Joyal J.-S., Talukdar S. (2021). Vitreous metabolomics profiling of proliferative diabetic retinopathy. Diabetologia.

[49] Zhou Y., Tan W., Zou J., Cao J., Huang Q., Jiang B., Yoshida S., Li Y. (2021). Metabolomics Analyses of Mouse Retinas in Oxygen-Induced Retinopathy. Investig. Ophthalmol. Vis. Sci..

[50] Zhou Y., Xu Y., Zhang X., Huang Q., Tan W., Yang Y., He X., Yoshida S., Zhao P., Li Y. (2021). Plasma levels of amino acids and derivatives in retinopathy of prematurity. Int. J. Med. Sci..

[51] Narayanan S.P., Xu Z., Putluri N., Sreekumar A., Lemtalsi T., Caldwell R.W., Caldwell R.B. (2014). Arginase 2 deficiency reduces hyperoxia-mediated retinal neurodegeneration through the regulation of polyamine metabolism. Cell Death Dis..

[52] Singh C., Benos A., Grenell A., Rao S., Anand-Apte B., Sears J.E. (2021). Hyperoxia Inhibits Proliferation of Retinal Endothelial Cells in a Myc-Dependent Manner. Life.

[53] Yamashita T., Nishimura K., Saiki R., Okudaira H., Tome M., Higashi K., Nakamura M., Terui Y., Fujiwara K., Kashiwagi K. (2013). Role of polyamines at the G1/S boundary and G2/M phase of the cell cycle. Int. J. Biochem. Cell Biol..

[54] Singh C., Tran V., McCollum L., Bolok Y., Allan K., Yuan A., Hoppe G., Brunengraber H., Sears J.E. (2020). Hyperoxia induces glutamine-fuelled anaplerosis in retinal Müller cells. Nat. Commun..

[55] Hellström A., Nilsson A.K., Wackernagel D., Pivodic A., Vanpee M., Sjöbom U., Hellgren G., Hallberg B., Domellöf M., Klevebro S. (2021). Effect of Enteral Lipid Supplement on Severe Retinopathy of Prematurity: A Randomized Clinical Trial. JAMA Pediatr..

[56] Löfqvist C.A., Najm S., Hellgren G., Engström E., Sävman K., Nilsson A.K., Andersson M.X., Hård A.L., Smith L.E.H., Hellström A. (2018). Association of Retinopathy of Prematurity with Low Levels of Arachidonic Acid: A Secondary Analysis of a Randomized Clinical Trial. JAMA Ophthalmol..

[57] Fu Z., Chen C.T., Cagnone G., Heckel E., Sun Y., Cakir B., Tomita Y., Huang S., Li Q., Britton W. (2019). Dyslipidemia in retinal metabolic disorders. EMBO Mol. Med..

[58] Tomita Y., Usui-Ouchi A., Nilsson A.K., Yang J., Ko M., Hellström A., Fu Z. (2021). Metabolism in Retinopathy of Prematurity. Life.

[59] Connor K.M., SanGiovanni J.P., Lofqvist C., Aderman C.M., Chen J., Higuchi A., Hong S., Pravda E.A., Majchrzak S., Carper D. (2007). Increased dietary intake of omega-3-polyunsaturated fatty acids reduces pathological retinal angiogenesis. Nat. Med..

[60] Fu Z., Lofqvist C.A., Shao Z., Sun Y., Joyal J.S., Hurst C.G., Cui R.Z., Evans L.P., Tian K., SanGiovanni J.P. (2015). Dietary ω-3 polyunsaturated fatty acids decrease retinal neovascularization by adipose-endoplasmic reticulum stress reduction to increase adiponectin. Am. J. Clin. Nutr..

[61] Miller J.W. (2013). Age-related macular degeneration revisited—Piecing the puzzle: The LXIX Edward Jackson memorial lecture. Am. J. Ophthalmol..

[62] Crabb J.W., Miyagi M., Gu X., Shadrach K., West K.A., Sakaguchi H., Kamei M., Hasan A., Yan L., Rayborn M.E. (2002). Drusen proteome analysis: An approach to the etiology of age-related macular degeneration. Proc. Natl. Acad. Sci. USA.

[63] Qi J.H., Bell B., Singh R., Batoki J., Wolk A., Cutler A., Prayson N., Ali M., Stoehr H., Anand-Apte B. (2019). Sorsby Fundus Dystrophy Mutation in Tissue Inhibitor of Metalloproteinase 3 (TIMP3) promotes Choroidal Neovascularization via a Fibroblast Growth Factor-dependent Mechanism. Sci. Rep..

[64] Ostergaard J.R. (2016). Juvenile neuronal ceroid lipofuscinosis (Batten disease): Current insights. Degener. Neurol. Neuromuscul. Dis..

[65] Palmer D.N., Fearnley I.M., Walker J.E., Hall N.A., Lake B.D., Wolfe L.S., Haltia M., Martinus R.D., Jolly R.D. (1992). Mitochondrial ATP synthase subunit c storage in the ceroid-lipofuscinoses (Batten disease). Am. J. Med. Genet..

[66] Chao J.R., Knight K., Engel A.L., Jankowski C., Wang Y., Manson M.A., Gu H., Djukovic D., Raftery D., Hurley J.B. (2017). Human retinal pigment epithelial cells prefer proline as a nutrient and transport metabolic intermediates to the retinal side. J. Biol. Chem..

[67] Du J., Yanagida A., Knight K., Engel A.L., Vo A.H., Jankowski C., Sadilek M., Tran V.T., Manson M.A., Ramakrishnan A. (2016). Reductive carboxylation is a major metabolic pathway in the retinal pigment epithelium. Proc. Natl. Acad. Sci. USA.

[68] Laíns I., Chung W., Kelly R.S., Gil J., Marques M., Barreto P., Murta J.N., Kim I.K., Vavvas D.G., Miller J.B. (2019). Human Plasma Metabolomics in Age-Related Macular Degeneration: Meta-Analysis of Two Cohorts. Metabolites.

[69] Mitchell S.L., Ma C., Scott W.K., Agarwal A., Pericak-Vance M.A., Haines J.L., Jones D.P., Uppal K., Brantley M.A. (2021). Plasma Metabolomics of Intermediate and Neovascular Age-Related Macular Degeneration Patients. Cells.

[70] Acar İ.E., Lores-Motta L., Colijn J.M., Meester-Smoor M.A., Verzijden T., Cougnard-Gregoire A., Ajana S., Merle B.M.J., de Breuk A., Heesterbeek T.J. (2020). Integrating Metabolomics, Genomics, and Disease Pathways in Age-Related Macular Degeneration: The EYE-RISK Consortium. Ophthalmology.

[71] Duncan J.L. (2005). Mouse models may provide new insight into the relation between cholesterol and age related macular degeneration. Br. J. Ophthalmol..

[72] Holoman N.C., Aiello J.J., Trobenter T.D., Tarchick M.J., Kozlowski M.R., Makowski E.R., De Vivo D.C., Singh C., Sears J.E., Samuels I.S. (2021). Reduction of Glut1 in the Neural Retina But Not the RPE Alleviates Polyol Accumulation and Normalizes Early Characteristics of Diabetic Retinopathy. J. Neurosci..

[73] Hammes H.P., Federoff H.J., Brownlee M. (1995). Nerve growth factor prevents both neuroretinal programmed cell death and capillary pathology in experimental diabetes. Mol. Med..

[74] Yun J.H., Kim J.M., Jeon H.J., Oh T., Choi H.J., Kim B.J. (2020). Metabolomics profiles associated with diabetic retinopathy in type 2 diabetes patients. PLoS ONE.

[75] Zhu X.R., Yang F.Y., Lu J., Zhang H.R., Sun R., Zhou J.B., Yang J.K. (2019). Plasma metabolomic profiling of proliferative diabetic retinopathy. Nutr. Metab..

[76] Sumarriva K., Uppal K., Ma C., Herren D.J., Wang Y., Chocron I.M., Warden C., Mitchell S.L., Burgess L.G., Goodale M.P. (2019). Arginine and Carnitine Metabolites Are Altered in Diabetic Retinopathy. Investig. Ophthalmol. Vis. Sci..

[77] Peters K.S., Rivera E., Warden C., Harlow P.A., Mitchell S.L., Calcutt M.W., Samuels D.C., Brantley M.A. (2021). Plasma Arginine and Citrulline are Elevated in Diabetic Retinopathy. Am. J. Ophthalmol..

[78] Sun Y., Zou H., Li X., Xu S., Liu C. (2021). Plasma Metabolomics Reveals Metabolic Profiling for Diabetic Retinopathy and Disease Progression. Front. Endocrinol..

[79] Mora-Ortiz M., Nuñez Ramos P., Oregioni A., Claus S.P. (2019). NMR metabolomics identifies over 60 biomarkers associated with Type II Diabetes impairment in db/db mice. Metabolomics.

[80] Patrick A.T., He W., Madu J., Sripathi S.R., Choi S., Lee K., Samson F.P., Powell F.L., Bartoli M., Jee D. (2020). Mechanistic dissection of diabetic retinopathy using the protein-metabolite interactome. J. Diabetes Metab. Disord..

[81] Yan W., Peng Y.-R., van Zyl T., Regev A., Shekhar K., Juric D., Sanes J.R. (2020). Cell Atlas of The Human Fovea and Peripheral Retina. Sci. Rep..

[82] Fligor C.M., Langer K.B., Sridhar A., Ren Y., Shields P.K., Edler M.C., Ohlemacher S.K., Sluch V.M., Zack D.J., Zhang C. (2018). Three-Dimensional Retinal Organoids Facilitate the Investigation of Retinal Ganglion Cell Development, Organization and Neurite Outgrowth from Human Pluripotent Stem Cells. Sci. Rep..

[83] Kim S., Lowe A., Dharmat R., Lee S., Owen L.A., Wang J., Shakoor A., Li Y., Morgan D.J., Hejazi A.A. (2019). Generation, transcriptome profiling, and functional validation of cone-rich human retinal organoids. Proc. Natl. Acad. Sci. USA.

